# Otologic and audiologic characteristics of type 2 diabetics in a tertiary health institution in Nigeria^☆^

**DOI:** 10.1016/j.bjorl.2015.10.016

**Published:** 2016-02-10

**Authors:** Stephen Oluwatosin Adebola, Micheal A. Olamoyegun, Olusola A. Sogebi, Sandra O. Iwuala, John A. Babarinde, Abayomi O. Oyelakin

**Affiliations:** a Ladoke Akintola University of Technology (LAUTECH) Teaching Hospital, Department of Otorhinolaryngology, Ogbomoso, Nigeria; b Ladoke Akintola University of Technology, Endocrinology, Diabetes and Metabolism Unit, College of Health Sciences, LAUTECH Teaching Hospital, Ogbomoso, Nigeria; c Olabisi Onabanjo University, College of Health Sciences, ENT Unit, Department of Surgery, Sagamu, Nigeria; d University of Lagos, Lagos University Teaching Hospital, and College of Medicine, Department of Medicine, Endocrinology, Diabetes and Metabolism Unit, Lagos, Nigeria

**Keywords:** Diabetes mellitus, Ear disease, Hearing impairment, Pure tone audiometry, Nigeria, Diabetes melito, Doença otológica, Deficiência auditiva, Audiometria tonal, Nigéria

## Abstract

**Introduction:**

This cross-sectional comparative study was carried out at the Diabetes outpatient clinic of LAUTECH Teaching Hospital (LTH) Ogbomoso, Nigeria.

**Objective:**

This study assessed patterns of otologic diseases and auditory acuity among type 2 diabetics and determinants of these findings among diabetics.

**Methods:**

Ninety-seven consenting patients with clinical diagnosis of diabetes mellitus (194 ears) were matched for age and sex with ninety non-diabetic patients (180 ears). These patients were screened using otoscopy and pure tone audiometry over a 6-month period.

**Results:**

The study reported a crude prevalence rate of 21.6% hearing loss in T2DM patients. The most common type of otologic disease that showed significant association with T2DM patients was otitis media with effusion (*p* = 0.027). T2DM was significantly associated with abnormal audiometric findings (*p* = 0.022), particularly sensorineural hearing loss (*p* = 0.022), of the moderate grade (*p* = 0.057). There were no differences of the audilogical findings for any particular ear, and no differential affectation of frequency range was observed. Coexisting hypertension and poor glycaemic control were significantly associated with aggravation of the hearing of the T2DM patients (*p* <  0.001, and *p* = 0.009 respectively).

**Conclusion:**

T2DM had appreciable effects on hearing acuity. T2DM was significantly associated with the type and the degree of the hearing loss. The need for screening of hearing acuity of T2DM patients, in order to detect early changes, and promptly offer an adequate management and remedial measures was emphasized in this study.

## Resumo

**Introdução:**

Este estudo de caso-controle foi realizado no Ambulatório de Diabetes do *LAUTECH Teaching Hospital* (LTH), em Ogbomoso, Nigéria.

**Objetivo:**

Este estudo avaliou os padrões de doenças otológicas e acuidade auditiva entre os diabéticos tipo 2 e os fatores determinantes desses achados entre os diabéticos.

**Método:**

Ao todo, 97 pacientes com diagnóstico clínico de diabetes melito (194 orelhas) deram seu consentimento e foram pareados por idade e sexo, com 90 pacientes não diabéticos (180 orelhas). Eles foram avaliados por otoscopia e audiometria tonal liminar por um período de seis meses.

**Resultados:**

O estudo relatou uma taxa de prevalência bruta de 21,6% de perda auditiva em pacientes com DM2. O tipo mais comum de doença otológica, afetando significativamente pacientes com DM2, foi otite média com efusão (p = 0,027). A DM2 foi associada com achados audiométricos alterados (p = 0,022), principalmente perda auditiva neurossensorial (p = 0,012), de grau moderado (p = 0,057). Não houve predileção dos efeitos da DM2 para uma das orelhas em particular, e também não houve efeito diferencial da faixa de frequência. A coexistência de hipertensão e controle glicêmico inadequado associou-se significativamente à piora da audição dos pacientes com DM2 (p < 0,001 e p = 0,009, respectivamente).

**Conclusão:**

A DM2 mostrou apresentar efeitos significantes na acuidade auditiva afetando significativamente o tipo e o grau da disacusia. Este estudo mostrou a necessidade de avaliação da acuidade auditiva de pacientes com DM2, a fim de detectar as alterações iniciais, e poder rapidamente oferecer medidas corretivas adequadas.

## Introduction

Diabetes mellitus is a chronic metabolic disorder characterized by hyperglycemia due to inadequate insulin secretion, ineffective action or a combination of these. Worldwide, the prevalence of diabetes is increasing although the rate varies from countries, races and religion. It has been estimated that the prevalence of diabetes will increase from present 382 million to 592 million by 2035.^1^ The vast majority of diabetes cases fall into 2 major etiopathogenetic categories, Type 1 diabetes which occurs as a result of absolute deficiency of insulin secretion and Type 2 diabetes which is caused by a combination of insulin resistance and a faulty compensatory insulin secretory response.^2^ Other categories include; Other specific types (caused by specific genetic defects, surgery, drugs); Gestational Diabetes Mellitus (GDM); Impaired Glucose Tolerance (IGT) and Impaired Fasting Glucose (IFG).^2^ Type 2 diabetes mellitus (T2DM) accounts for 80%–90% of all cases of diabetes and is closely related to obesity among other risk factors. It is a multisystem disorder with a propensity to affect the cardiovascular system, and produces varying chronic microvascular and macrovascular complications. Amongst the complications, hearing loss remains one of its most-distressing and least understood phenomenon. Thus researchers have proposed various hypotheses including micro-angiopathy and neuropathy to explain the complication.^3^, ^4^, ^5^ Infact, studies have demonstrated that the micro-angiopathy in Type 2 Diabetes Mellitus (T2DM) involves mostly the cochlear with associated degeneration of the stria vascularis and cochlear outer hair cells.^6^, ^7^

The pattern of hearing loss in diabetes has been shown in many studies^8^, ^9^, ^10^, ^11^, ^12^, ^13^, ^14^ to be moderately severe in magnitude, progressive in nature, and bilateral in occurrence and may be irreversible. The prevalence of hearing loss in diabetics in Nigerian population has not been studied extensively.^9^, ^15^ This study was undertaken to describe the pattern of otologic diseases and auditory acuities in T2DM patients comparing this with those of non-diabetics and to also explore the determinants of these patterns. This is important because type 2 diabetes mellitus is a public health problem, which significantly and negatively affect the quality of life when complicated with hearing loss.^15^

## Methods

### Study design

This cross-sectional and comparative study was carried out on patients attending the Endocrinology and General outpatient clinics in Ladoke Akintola University of Technology Teaching Hospital (LTH) Ogbomoso, Nigeria. One hundred and 87 patients were divided into two groups based on their diabetes status; Group I consisted of 97 patients diagnosed with type 2 diabetes (T2DM), attending the endocrinology clinic and the group two consisted of 90 non-diabetic patients attending the general outpatient clinic, which were matched for age and sex with the study group. The study was carried out over a 6 month period from December 2013 to May 2014.

The study was approved by the institutional Ethics committee of the Ladoke Akintola University of Technology Teaching Hospital (LTH) Ogbomoso, Nigeria. Appropriate sample size was determined according to the methodology of other published and relevant studies.^14^

Sampling technique: Consecutive adult patients attending each of these clinics were approached as potential subjects. The purpose, nature, significance of the study was explained to each of the subjects; those that consented were recruited as subjects. The main inclusion criteria for group one were: known diabetic patients who had attended at least 2 visits at outpatient diabetic clinics, aged ≥ 30 years with a confirmed diagnosis of T2DM based on WHO criteria of Fasting Plasma Glucose (FPG) of ≥ 126 mg/dL (7 mmoL/L), regarded as the test subjects. For the patients of the group two, normal blood glucose level (taken as FPG  <  110 mg/dL (6.1 mmoL/L) served as controls. A diagnosis of hypertension was made in patients with systolic blood pressure > 140 mmHg and diastolic blood pressure > 90 mmHg.

Patients with history of consumption of ototoxic drugs within three months, previous history of ear surgeries, recent infections in the ear, nose and throat, history suggestive of exposure to noise induced hearing loss were excluded. Data were generated from the information extracted from the participant by the researchers using an interviewer-administered questionnaire. The information obtained included socio-demographic parameters like the age last birthday, sex, social classes^16^ and occupation of each participant.

The weight measured in kilograms (kg) of the subjects were taken by using Omron HN-283 digital body weight scale, and the height measurements in metres (m) were taken by Seca Leicester standiometer. The Body Mass Index (BMI) was calculated as weight in kg/(height in metres)^2^. The ranges of the BMI were; underweight (< 18.0), normal weight (18.1–24.9), overweight (25.0–29.9), Obese (> 30.0), Types 1 to 3 (30.0–34.5, 35.0–39.9 and > 40 respectively).

Plasma glucose levels were measured for each participant, after withdrawing 2 mls of venous blood from each individual after an overnight fast of at least eight hours. The values obtained was using in classifying the patients into either of the groups. The other laboratory data determined in diabetic patients was glycated haemoglobin (HbA1c) using Point of Care machine (In 2 It^R^). HbA1c < 7% was considered as good glycaemic control and ˃ 7% as poor glycaemic control.

### Otoscopic and audiologic screening

Otoscopic examination was performed on both ears by a Consultant Otolaryngologist using a pneumatic otoscope. The findings of the outer ear, external auditory canal and the status of the tympanic membrane were recorded. Only one major diagnosis made from each ear was recorded. Pneumatic otoscopy was carried out to test mobility of the tympanic membrane using the Welch Allyn 3.5 v pneumatic otoscope (Model 20200).

Diagnostic pure tone audiometry was performed in a sound-proof booth with ambient noise of < 45 dB using a calibrated 2 channel audiometer (MA 53, Maico Inc.) by the same audiologist on all the subjects. Air conduction thresholds measured at frequencies 250, 500, 1000, 2000, 4000, 6000, 8000 Hertz, while the bone conduction thresholds were measured at the frequencies 250, 500, 1000, 2000, 4000 Hz for each ear separately. The method used was based on the American Speech Hearing Association (ASHA) guidelines for manual pure tone audiometry.^15^

### Statistical analysis

All statistical analysis were done using SPSS statistical package for Windows, version 18 (SPSS Inc., Chicago, IL). The results were presented in simple charts and tables as appropriate descriptive analyses. Comparative analyses of the different variables were performed between the T2DM patients and controls. Differences between categorical variables were explored using the Chi- square test, while those between continuous variables were explored using student t-test. Factors associated with hearing impairment in the diabetics were also explored. All analyses were done with statistical significant level set at *p*  <  0.05.

## Results

**Figure 1:**
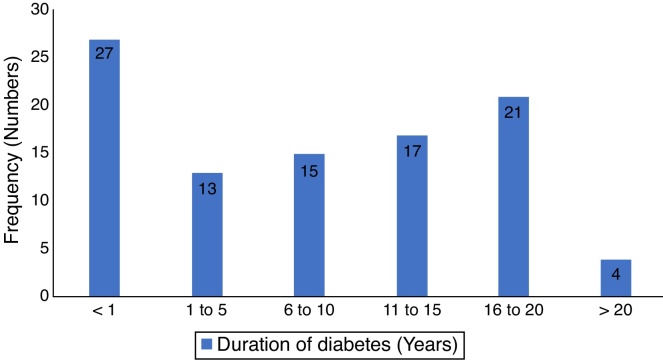
Showing the duratiom of diabetes mellitus in subjects.

There were one hundred and eighty seven patients comprising of 97 test-subjects with type 2 diabetes (T2DM) and 90 control subjects without diabetes who participated in this study. The gender distribution among T2DM (44 males and 53 females) and the controls (40 males and 50 females) were similar (*p* = 0.899). The low socio economic class (groups IV and V) constituted 62.9% and 52.1% for the test subjects and controls respectively, with no statistically significant difference (*p* = 0.693). The body-mass index (BMI) of the participants showed that 80.4% of diabetics had BMI ≥ 30.0 compared to 56.7% of controls (*p* < 0.001). There were no significant differences in the otoscopic findings such as cerumen impaction and tympanic membrane perforations between the two groups of patients (*p* = 0.781 and 0.083 respectively). However, a significant higher number of T2DM patients had OME (*p* = 0.027). Other socio-demographic and clinical characteristics of participants are as seen in Table 1. The mean duration of diabetes was 7.6 years (S.D = 6.4), while diagnosis  <  1 year period was the most prevalent, 27.8%, the details of the distribution is in Fig. 1.

**Table 1:** Socio-demographic and clinical characteristics of the participants.

	Diabetics	Controls		
Parameter	n = 97 (%)	n = 90 (%)	χ^2^	*p*-value
*Gender of participants*				
Male	44 (45.4)	40 (44.4)		
Female	53 (54.6)	50 (55.9)	0.016	0.889

*Age range (years)*				
30–39	10 (10.3)	10 (11.1)		
40–49	18 (18.6)	16 (17.8)		
50–59	11 (11.3)	12 (13.3)		
60–69	35 (36.1)	30 (33.3)		
70–79	12 (12.1)	12 (13.3)		
≥ 80	11 (11.3)	10 (11.1)		
Mean ± SD	58.9 ± 14.95	58.8 ± 14.71		0.997

*Socio-economic class*				
I	8 (8.2)	9 (10.0)		
II	11 (11.3)	14 (15.6)		
III	17 (17.6)	20 (22.2)		
IV	28 (28.9)	21 (23.3)		
V	33 (34.0)	26 (28.9)	2.234	0.693

*Body Mass Index (BMI)*				
Healthy weight	19 (19.6)	39 (43.3)		
Overweight	27 (27.8)	31 (34.4)		
Obesity type 1	15 (15.5)	11 (12.3)		
Obesity type 2	36 (37.1)	9 (10.0)	23.759	<0.001^a^

*Otoscopic findings*	n = 194 (%)	n = 180 (%)		
Normal	99 (51.0)	116 (64.4)	1.344	0.246
Impacted Cerumen auris	27 (13.9)	25 (13.9)	0.077	0.781
Otitis Media with Effusion	38 (19.6)	21 (11.7)	4.898	0.027^a^
Perforated Tympanic membrane	30 (15.5)	18 (10.0)	3.000	0.083

a Statistically significant.

Abnormal pure tone audiometry (PTA) findings were present in 21.5% (21) and 8.9% (8) of the T2DM and controls respectively, which was statistically significant (*p* = 0.016). The affectation of the ears of participants with abnormal PTA findings was unilateral in five of the twenty-one T2DM patients (5/21, 23.8%) and six of the ten controls (6/10, 75.0%). The number of participants with abnormal PTA, with bilateral ear involvement found in T2DM patients was significantly higher than in controls (*p* =  <  0.001). The most prevalent type of hearing loss recorded among T2DM patients (in 61.9%) was sensorineural hearing loss which was significantly different (*p* = 0.012) from the 37.5% recorded among the controls. The degree of hearing loss most common amongst T2DM patients and controls in the study was moderate hearing loss, 38.0% (8/21) and mild hearing loss, 37.5% (3/8) respectively, which were not statistically significant (Table 2).

**Table 2:** Audiologic characteristics of the participants.

	Diabetic patients	Controls		
Parameter	n = 97 (%)	n = 90 (%)	χ^2^	*p*-value
*Abnormal PTA findings*				
No	76 (78.4)	82 (91.1)		
Yes	21 (21.5)	8 (8.9)	5.828	0.016*

*Level of involvement*				
Unilateral	5 (23.8)	6 (75.0)	0.091	0.763
Bilateral	16 (76.2)	2 (25.0)	10.889	< 0.001*

*Type of hearing loss*				
Conductive HL	3 (14.2)	2 (25.0)	0.250	0.650
Sensori-neural HL	13 (61.9)	3 (37.5)	6.250	0.012^a^
Mixed HL	5 (23.9)	3 (37.5)	0.500	0.480

*Degree of the hearing loss*				
Mild (26–40 db)	4 (19.0)	3 (37.5)	0.143	0.705
Moderate (41–60 db)	8 (38.0)	2 (25.0)	3.600	0.057
Severe (61–80db)	5 (24.0)	2 (25.0)	1.286	0.257
Profound (> 80 db)	4 (19.0)	1 (12.5)	1.800	0.180

PTA, Pure Tone Audiometry; HL, Hearing Loss.

a Statistically significant.

**Figure 2:**
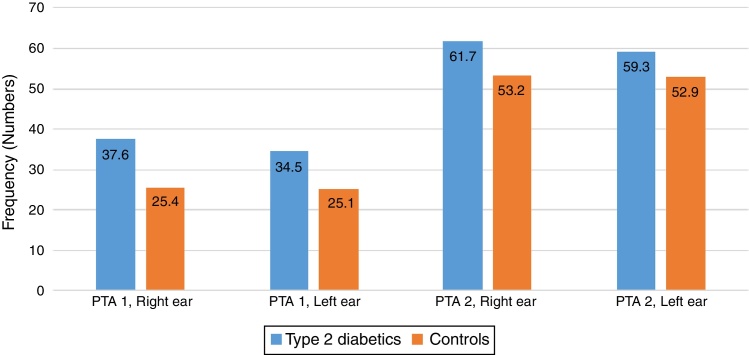
Pure tone audiometric averages at diferente frequency ranges according to subject category.

There were some differences noted between the T2DM group and the control group in the two pure tone average (PTAv) groups computed: PTAv-1 (0.5, 1, 2 kHz) and PTAv-2 (4, 6, 8 kHz). Diabetics needed more sound intensity for the detection of PTA threshold as compared with controls (Fig. 2). The average threshold differences ranged from 8.5 to 12.2 decibels (dB) in the right ear, and from 6.4 to 9.4 dB in the left ear. These differences were not statistically significant.

A total of 21 participants with T2DM had hearing impairment (bilateral in 16 and unilateral in 5) out of a total of 97 T2DM patients screened. This gave a crude prevalence rate of 21.6%. Among the subjects with T2DM alone abnormal PTA results were found in 28.6% while it was found among 71.4% of patients with T2DM co-existing with hypertension (*p*  <  0.001). Comparing the audiograms between patients with good and poor glycaemic control, revealed significant differences in between the two groups of patients (*p* = 0.009) in Table 3.

**Table 3:** Showing the relationship between diabetic parameters and hearing impairment of participants.

Diabetes mellitus parameters	Audiologic parameters		χ^2^	*p*-value
	Normal PTA n = 76 (%)	Abnormal PTA n = 21 (%)		
*Co-Morbidities*				
Diabetes mellitus alone	33 (43.4)	6 (28.6)		
Diabetes mellitus with hypertension	43 (56.6)	15 (71.4)	13.517	< 0.001^a^

*Glycemic Control*				
Good control (HbA1c < 7%)	52 (68.4)	12 (57.2)		
Poor control (HbA1c < 7%)	24 (31.6)	9 (42.8)	6.818	0.009^a^

a Statistically significant.

## Discussion

The study found that the main otological disease associated with T2DM patients in our environment was otitis media with effusion. There were significant impacts of T2DM on the audiological characteristics namely abnormal audiometric shapes, sensorineural type of hearing loss, involving the two ears. T2DM did not differentially affect any frequency range and had no effect on the degree of hearing loss. Hearing impairment in T2DM patients were aggravated by co-existing hypertension and poor glycaemic control.

While the socio-demographic characteristics of the two categories of patients were generally similar, significantly more of the diabetic patients were obese or at least had unhealthy weights similar to previous studies.^4^, ^17^ The preponderance of the low socio-economic class, 62.9% and 52.1% in both T2DM patients and controls in the study suggest the peculiarities of this class of individuals. The level of education in this class tend to be low as compared with the higher classes and added to this fact is the tendency to take diets that may not be balanced nutritionally, predisposing to obesity. Obesity had been established as one of the risk factors for T2DM,^18^ and maintenance of healthy weight has been mentioned among the ways for preventing development of T2DM.^17^, ^19^ Furthermore, weight reduction remains one of the key lifestyle modification strategies that has been deployed in the management of T2DM.^20^, ^21^

While the spectrum of otologic diseases found in this study was varied, it was only otitis media with effusion (OME) that was found to be significantly associated with T2DM. This finding is corroborated by previous studies in developing countries,^22^ where immune-compromised state has been implicated as a predisposing factor to developing chronic ear disease especially with anaerobic bacteriology in middle ear isolates. Nasal mucociliary action tends to be decreased in diabetics, which might predispose to OME.^23^ The study of Lee, Sun Kyu^24^ which examined the relationship between paediatric obesity and OME, noted that obesity results in altered cytokine expression, gastroesophageal reflux disease and fat accumulation, which may eventually result in OME. There may be need for further research on the relationship between T2DM and OME, to explore and fill the obvious gap in knowledge concerning these phenomena.

Two otological diseases, namely otomycosis and necrotizing (malignant) otitis externa (NOE) have been reportedly associated with diabetes were not found in this study. Otomycosis is a fungal infection which thrives in the ear when the systemic immunity is generally compromised especially in poor glycaemic control. The common practice of many of our patients instilling off-the-shelf anti-microbial and antifungal ear drops into the ear canals which might inadvertedly treat otomycosis, and may be responsible for its absence in this population. It is also possible that our patients’ diabetic control was not bad, especially when the simple majority (27.8%) among them were diagnosed within one year of noticing the symptoms. This may also be responsible for the absence of NOE in this study. However, it is possible that some of these pathologies were missed or misdiagnosed as the special tests required for their diagnoses like newer molecular detection methods available as pre-packaged kits (yeast star, auxocoloretic),^25^ immunoassay with monoclonal antibodies, and polymerase chain reaction (PCR) were not carried out in the study.

Significantly more of the T2DM patients in this study were found to have abnormal audiologic characteristics, particularly of the sensorineural type that affected the two ears. The systemic nature of the disease will tend to affect the two ears, and there might not be a lateralization to a particular ear. This is in tandem with findings from other researchers.^26^ In patients with diabetes, two possible mechanisms had been muted which includes microangiopathic changes involving all the major blood vessels including that of the inner ear in T2DM patients^7^, ^11^ Secondly there could be primary neuropathy of the cochlear nerve leading to retro-cochlear hearing loss.^12^ The hearing impairment in this study significantly affected both ears. Ciorba et al.^8^ in a large cohort study that evaluated inner ear changes resulting from micro-vascular disease in T2DM patients, however reported that the association of SNHL with T2DM was not conclusive.

Some previous studies have not identified any relationship between duration of diabetes, presence and degree of hearing loss.^12^, ^13^, ^14^ Similarly in this study, we found that T2DM did not differentially affect any frequency range and had no effect on the degree of hearing loss. Systemic diseases like presbycusis, and noise induced hearing loss have been reported to differentially affect the high frequency tones,^27^ while Frisina et al., in a study in USA, found the greatest hearing deficits at the lower frequencies among patients with T2DM.^28^ Mozaffari et al.^29^ in the study carried out in Iran amongst the non-elderly population (< 60 years old), however noted that age at onset and duration of diabetes mellitus were predisposing factors to acquiring hearing loss in diabetics. It is obvious in this study that T2DM significantly affected hearing of the subjects. Thus certain actions may be necessary especially as hearing impairment itself can constitute a major morbidity. The management of T2DM will require a multidisciplinary approach involving the diabetologist, the dietitian, the otologist, audiologist and possibly the neurologist. We propose that screening of hearing acuities with diagnostic pure tone audiometry be performed at diagnosis of T2DM, and at least six-monthly intervals on the initial results of the tests. This will allow early detection of auditory anomalies, permit prompt and adequate management to be offered and remedial measures instituted as appropriate.

Hearing impairment in T2DM patients are aggravated by co-existing hypertension and poor glycaemic control. This is in keeping with findings of Duck et al.,^30^ whose study supported the hypothesis that the presence of end-organ damage in diabetics, tends to be exacerbated in the co-existing hypertension. However the impact of hypertension in T2DM patients in this study must be made cautiously, as other known and reported confounders like hypercholesterolemia, heavy smoking, and genetic predisposition^18^ were not controlled for. Furthermore, as reported in some other studies^9^, ^12^, ^31^ we found that hearing impairment in T2DM patients were also aggravated by poor glycaemic control. Prolonged accumulation of products of glycation in the inner ear, especially the outer hair cell function has been attributed to be responsible for the hearing impairment associated with poor glycaemic control among T2DM patients. However, this explanation is not definitive, especially as some other studies had not found a significant association between these two phenomena,^32^ The exact mechanisms of glycosylation on the hair cell functions is the subject of some on-going experimental research studies with animal models.

The study was limited by its hospital-based nature, and the fact that other confounders and co-morbidities were not subjected to control. A larger population-based epidemiological study might be necessary for better elucidation of this important subject.

## Conclusion

In conclusion, this study found that T2DM had appreciable effects on hearing acuity, showing a significant association between the disease and the type and the degree of the hearing loss. There is a need for screening of hearing acuity of T2DM patients, in order to detect the changes early, offering a prompt adequate management and remedial measures as appropriate.

## Conflicts of interest

The authors declare no conflicts of interest.
